# Myosin-X Acts Upstream of L-Plastin to Drive Stress-Induced Tunneling Nanotubes

**DOI:** 10.3390/cells15030224

**Published:** 2026-01-24

**Authors:** Ana Ramirez Perez, Joey Tovar, Karine Gousset

**Affiliations:** Biology Department, College of Science and Mathematics, California State University Fresno, Fresno, CA 93740, USA

**Keywords:** tunneling nanotubes, TNTs, myosin-X, Myo10, L-plastin, LCP1, plastin-2

## Abstract

**Highlights:**

**What are the main findings?**

- Myo10 acts upstream of L-plastin in stress-induced TNT formation, defining a unidirectional Myo10 to L-plastin regulatory axis.
- L-plastin is a key structural effector for TNT abundance: upregulating L-plastin increases TNT-connected cells, while downregulating L-plastin reduces TNT-connected cells (with Myo10 and L-plastin co-localizing along TNTs and proto-TNTs).

**What are the implications of the main findings?**

- The Myo10 to L-plastin axis provides a mechanistic link between stress signaling and stable TNT formation by coupling Myo10-driven protrusion initiation to L-plastin–mediated actin-bundle stabilization.
- This highlights a potentially targetable pathway to modulate TNT networks in disease (e.g., cancer resilience/chemoresistance) or to enhance beneficial TNT-dependent transfer (e.g., mitochondrial rescue in injured/stressed cells).

**Abstract:**

Tunneling nanotubes (TNTs) are thin, actin-based intercellular bridges that enable long-range communication during cellular stress; yet the molecular pathway controlling their formation remains unclear. Here, using gain- and loss-of-function approaches in Cath. a-differentiated (CAD) neuronal cells, we identified a unidirectional regulatory pathway in which myosin-X (Myo10) functions upstream of the actin-bundling protein L-(LCP1) to drive TNT formation. Using Western blotting and fluorescence microscopy, we determined that overexpression of L-plastin significantly increased the proportion of TNT-connected cells, whereas L-plastin downregulation reduced TNT formation, demonstrating that L-plastin is both sufficient and necessary for maintaining normal TNT abundance. Having previously shown that Myo10 is required for TNT formation in CAD cells, we asked whether the relationship is reciprocal. Overexpression/downregulation of L-plastin had no effect on Myo10 protein levels. Conversely, Myo10 downregulation decreased endogenous L-plastin by ~30%, and Myo10 overexpression elevated L-plastin expression and TNT number, demonstrating that Myo10 acts as an upstream regulator of L-plastin. Dual-color 3D imaging revealed co-localization of Myo10 and L-plastin along TNT shafts and filopodia-like precursors (Proto-TNTs). Together, these findings demonstrate that Myo10-dependent TNT formation requires the bundling protein L-plastin, providing a framework for how stress-induced signaling cascades couple TNT initiation to actin-core stabilization during stress and disease.

## 1. Introduction

Tunneling nanotubes (TNTs) are long, thin, actin-based membrane tubular structures that connect distant cells and enable direct intercellular transfer of organelles, vesicles, pathogens, and signaling molecules over tens to hundreds of micrometers [1,2,3]. First described in 2004 in neuronal PC12 cells [1,4], TNTs have since been observed in diverse cell types, including neurons, immune cells, cardiomyocytes, and cancer cells, where they mediate physiological processes such as mitochondrial rescue during oxidative stress or hypoxia, immune surveillance, and developmental signaling [5,6,7,8]. In pathology, TNTs facilitate the spread of misfolded proteins in neurodegenerative diseases [9], HIV-1 and prion transmission [10,11], and oncogenic cargo (e.g., mutant proteins, oncomiRs, or mitochondria) that promotes tumor progression, chemoresistance, and metabolic reprogramming of the microenvironment [12,13,14]. The dual role of TNTs in homeostasis and disease has made their formation and regulation an area of intense interest, yet the molecular pathway that governs TNT formation remains incompletely understood.

TNT formation is a multistep process that often begins when cellular stress is associated with the emergence of filopodia-like, actin-rich precursors, which we refer to as proto-TNTs. Multiple forms of stress—including mitochondrial damage, oxidative stress, hypoxia, therapy-induced stress, and elevated ROS/mtROS—have been shown to stimulate TNT formation and promote mitochondrial transfer as a rescue mechanism [5,15,16,17,18] Ca^2+^ signaling is also a major upstream trigger: activation of the Wnt/Ca^2+^–CaMKIIβ pathway enhances TNT formation and TNT-mediated cargo transfer [19], and Ca^2+^–calmodulin-dependent activation of Myo10, originally characterized in filopodia [20,21], is consistent with the Myo10-dependent TNT formation observed in neuronal CAD cells [22]. Once initiated, membrane protrusion and elongation rely heavily on Myo10 [22], an unconventional motor protein with cargo-transport functions. Myo10 dimerizes upon Ca^2+^–calmodulin binding, localizes to PIP_3_-rich membrane patches through its PH domains, and drives barbed-end-directed extension while transporting β-integrins and other adhesion receptors to protrusion tips [20,22,23,24]. Integrin transport by Myo10 supports both inside-out integrin activation and, upon ligand engagement, outside-in PI3K signaling, establishing a positive-feedback loop that amplifies local PIP_3_ and sustains protrusion [25,26,27,28]. Knockout or silencing of Myo10 significantly decreases TNT initiation in multiple systems, confirming its non-redundant role [22,29,30].

Once elongated, TNTs require robust parallel actin bundling for mechanical stability against membrane tension and shear forces. Fascin and Eps8 contribute bundling in some contexts [31,32], but L-plastin (plastin-2, LCP1), although originally described as leukocyte-specific, is frequently ectopically expressed in solid tumors and neuronally derived cell lines [33,34,35,36]. Consistent with this, we detect robust L-plastin expression in CAD cells.

L-plastin activity is tightly regulated: PIP_2_ and Ca^2+^ binding to its headpiece and EF-hands maintain an inactive conformation, whereas PI3K-mediated PIP_2_ depletion and SGK-dependent phosphorylation at Ser5 markedly increase F-actin affinity and bundling efficiency [37,38,39,40,41,42]. Because PI3K converts PIP_2_ to PIP_3_, the same PIP_3_-enriched membrane patches that recruit and activate Myo10 at protrusion tips could also be expected to favor L-plastin activation by simultaneously relieving PIP_2_-mediated inhibition and engaging SGK kinases downstream of PI3K. In this way, L-plastin acts as a bundling effector at the distal end of the Myo10–PI3K signaling axis, stabilizing actin filaments within TNT shafts. Our unpublished LCM-derived proteomic TNT data—using a method we developed in 2019 [43,44,45]—as well as recent proteomic and functional studies have placed L-plastin inside TNTs induced by hydrogen peroxide (H_2_O_2_) and show its upregulation correlates with increased TNT abundance in preneoplastic and transformed cells [46]; yet whether L-plastin is merely permissive or tightly regulated during TNT formation has remained unknown.

Here we identify Myo10 as an upstream regulator of L-plastin in stress-induced TNT formation. Using gain- and loss-of-function approaches in CAD neuronal cells, we show that Myo10 levels and L-plastin protein levels change in parallel—Myo10 overexpression increases L-plastin, whereas Myo10 down regulation decreases L-plastin expression—while L-plastin manipulation has no reciprocal effect on Myo10. Interestingly, altering L-plastin levels alone is sufficient to modulate TNT abundance. L-plastin overexpression increases the number of cells connected with TNTs, while L-plastin depletion decreases the proportion of TNT-connected cells. Together with our previous demonstration that Myo10 controls TNT abundance in CAD cells [22], these findings position Myo10 upstream of L-plastin in a pathway governing TNT formation. Combined with co-localization of both proteins along TNT shafts and proto-TNTs, these findings define a unidirectional Myo10 to L-plastin axis. This work begins to define how stress-induced signaling cascades couple protrusion initiation to actin-bundle stabilization during TNT formation.

## 2. Materials and Methods

### 2.1. Cell Lines, Antibodies, Transfection and Transduction

Mouse neuronal CAD cells (Sigma, St. Louis, MO, USA, 08100805) were cultured in Opti-MEM, glutamate reduced media (Life Technologies, Carlsbad, CA, USA, 51985-034) and supplemented with 10% Fetal Bovine Serum (FBS) (BioWest, Riverside, MO, S1620).

Transient transfections were performed using Lipofectamine 3000 (Invitrogen, Carlsbad, CA, USA, L3000008) in accordance with the manufacturer’s instructions. The GFP-tagged human L-plastin construct (pEGFP-N1-L-plastin) was provided by Dr. Ralph Boettcher (Max Planck Institute, Munich, Germany), and the bovine GFP-Myo10 plasmid by Dr. Richard E. Cheney (UNC, Chapel Hill, NC, USA).

For Western blot analyses and immunofluorescence, the primary antibodies used were the following: mouse anti–L-plastin (SCBT, Dallas, TX, USA, sc-133218), rabbit anti-Myo10 (Sigma, HPA024223), and Anti-GFP, N-terminal antibody produced in rabbit (Sigma, G1544). For Western blots, the secondary antibodies used were the following: StarBright Blue 700 Goat Anti-Rabbit IgG (Biorad, Hercules, CA, USA, 12004162) or StarBright Blue 700 Goat Anti-Mouse IgG, (Biorad, 12004159). For Immunofluorescence experiments, the secondary antibodies used were Goat anti-Rabbit IgG (H + L) Cross-Adsorbed Secondary Antibody, Alexa Fluor 546; or Rabbit anti-Mouse IgG (H + L) Cross-Adsorbed Secondary Antibody, Alexa Fluor 546 (Fisher Scientific, Pittsburg, PA, USA). For down regulation experiments, Myo10 shRNA (SCBT, sc-43242-V), L-plastin shRNA (SCBT, sc-43209-V), control shRNA lentiviral particles (SCBT, Dallas, TX, USA, sc-108060), Polybrene (sc-134220) reagent and puromycin dihydrochloride (Santa Cruz, sc-108071) were from Santa Cruz Biotechnology, Inc., and were used according to the manufacturer’s instructions. Additional reagents included tetramethylrhodamine-conjugated Wheat Germ Agglutinin, WGA-Oregon green or WGA-350 (Invitrogen, Carlsbad, CA, USA, cat # W7024), and hydrogen peroxide solution (30% *w*/*v*, Fisher Scientific, Pittsburg, PA, USA, cat # BP2633500).

### 2.2. Transient Transfection of GFP-L-Plastin and GFP-Myo10 in CAD Cells and TNT Counting

CAD cells numbering 100,000 were seeded in a 12-well plate supplemented with CAD media. Cells were transfected for 24 h using Lipofectamine 3000 (Invitrogen, L-3000-008) according to the manufacturer’s protocol. Briefly, 2 μg of plasmid DNA was diluted in 1.5 μL of Lipofectamine 3000 reagent in 100 μL of Opti-MEM together with 2.0 μL of P3000 reagent in 100 μL of Opti-MEM. Transfection complexes were incubated with cells for 24 h at 37 °C. Cells were then fixed by adding 200 μL of fix1 solution (2% PFA, 0.05% glutaraldehyde and 0.2 M HEPES in PBS) on top of cells in 500 μL media for 5 min, followed by a 15 min incubation in fix1 at room temperature (RT). Cells were then incubated for an additional 15 min incubation with fixative solution 2 (4% PFA and 0.2 M HEPES in PBS). Following fixation, cells were washed 3 times for 5 min in 1× PBS. Coverslips were incubated with Wheat Germ Agglutinin Texas Red TM-X (WGA) (1:100) (Invitrogen, W21405) in 1× PBS for 10 min at RT in the dark. After staining, coverslips were washed with 1× PBS 3 times for 5 min each. Finally, the coverslips were mounted onto glass slides using Aqua-Poly Mount. Mounted slides were subsequently stored at 4 °C until imaging. For each antibody, high resolution images were acquired using a widefield inverted Leica microscope (DMI3000, Leica Microsystems, Chicago, IL, USA) controlled by Metamorph acquisition software version 7.8.13.0 (Molecular Devices, San Jose, CA, USA), a 63×/1.25 oil objective, and a Leica DFC300 FX camera, Leica Microsystems Chicago, IL, USA. For each condition, Z-stacks were acquired in order to identify and quantify cells connected by TNTs.

All TNT quantification assays were performed under blinded conditions to minimize observer bias. TNT counts were assessed in 5 independent experiments for statistical comparison.

### 2.3. Lentiviral Particle Transduction and TNT Counting

CAD cells were seeded at the density of 40,000 cells per well in a 12-well plate and incubated for 24 h to approximately 50% confluency. The transduction process was performed according to the manufacturers’ protocol (SCBT, Dallas, TX, USA). Briefly, to generate stable clones, cells were exposed to lentiviral particles encoding control shRNA (SC-108080, SCBT, Dallas, TX, USA), L-plastin shRNA (SC-43209-V, SCBT, Dallas, TX, USA) or Myo10 shRNA (SC-43242-V, SCBT, Dallas, TX, USA) in the presence of Polybrene (SCBT, Dallas, TX, USA), which enhances viral transduction. Twenty-four-hour post-transduction, the culture medium was replaced with fresh growth medium. To select stably transduced cells, culture was maintained in medium containing 5 μg/mL puromycin dihydrochloride (SCBT, Dallas, TX, USA, sc-108071). Non-transduced cells were eliminated by puromycin selection, and surviving clones were expanded. Western blot analysis was used to evaluate the efficiency of gene silencing and to select clones exhibiting robust knockdown of L-plastin or Myo 10. Selected clones were maintained under continuous puromycin selection to ensure stable shRNA expression.

TNT counting was done as described in the section above.

### 2.4. Immunofluorescence Microscopy

To test whether endogenous L-plastin localizes within TNTs, 100,000 CAD cells were seeded onto glass coverslips in a 12-well tissue culture plate and incubated overnight in CAD media. To induce stressed-TNTs, the cells were treated with 100 uM H_2_O_2_ for 5 min, then fixed. Cell fixation: cells plated on coverslips overnight were fixed by adding 200 μL of fix1 (4% PFA and 0.2 M HEPES in PBS) dropwise to the cells in 500 μL media for 5 min. After 5 min, the solution was removed and cells were incubated for an additional 15 min in fix1 solution, followed by fix2 solution containing 5 mM DTBP and 25 mM HEPES in PBS for another 15 min. For cell membrane labeling, cells were incubated with WGA-Oregon Green 488 (Invitrogen, W7024) at a 1:100 dilution in PBS. Cells were permeabilized for 10 min with 0.1% Triton-X100, washed 3 times for 5 min with 1× PBS and blocked for 1 h using 5% goat serum (Jackson ImmunoResearch Lab. Inc., West Grove, PA, USA, 005-000-121) in PBS. Cells were incubated overnight at 4 °C with primary antibody against L-plastin and washed 3 times for 5 min with 1× PBS. The cells were then incubated for 50 min at RT in the dark with Alexa-546 anti-mouse (Invitrogen, A11030) in 5% goat serum. The cells were washed 3 more times for 5 min with 1× PBS and the coverslips were mounted using Aqua-Poly Mount (Polysciences Inc., Warrington, PA, USA, 18606-20). Negative controls: cells were permeabilized with Triton X-100 and incubated only with secondary antibodies to ensure that under our experimental conditions, we did not have nonspecific fluorescent signals from the secondary antibodies used. Z-stack confocal images for both experimental and control groups were acquired using an ANDOR BC43 Benchtop Confocal Microscope (Oxford Instruments, Andor Technology Inc, Concord, MA, USA) with a 60× Plan Apo LD Oil Objective (1.42NA 0.15WD) and its Fusion software (2.5.0.218 Benchtop) (Oxford Instruments, Andor Technology Inc, Concord, MA, USA). Bottom Z-stacks corresponding to adhering filopodia were not taken into consideration, to focus on TNTs (i.e., structures connecting cells that do not become attached to the substratum). Images were deconvolved using the Fusion software and any further image processing, such as “spot rendering” or “filament tracer”, was obtained using the Imaris imaging software (version 10.2.0) (Oxford Instruments, Andor Technology Inc, Concord, MA, USA).

### 2.5. Cell Lysis, Gel Electrophoresis, and Western Blotting

Transfected and/or Transduced CAD cells were lysed in RIPA buffer (10 mM Tris-HCl, pH 8.0; 1 mM EDTA; 0.5 mM EGTA; 1% Triton X-100; 0.1% sodium deoxycholate; 0.1% SDS; 140 mM NaCl) supplemented with Protease Inhibitor 100× (ThermoScientific, 1861278). Protein concentrations were measured with a Bradford Assay (Bio-Rad, Hercules, CA, USA, 5000006). Equal amounts of protein (10 micrograms (μg) per sample) were prepared using a 4× Laemmli buffer (Bio-Rad, 1610747) containing beta-2-mercaptoethanol (MP Biomedical, Irvine, CA, USA, 194834) and denature by boiling at 100 °C for 5 min prior to gel electrophoresis.

Protein samples (10 μg per lane) were loaded onto 8% Bis-Tris stain free polyacrylamide gels containing 2,2,2,trichloroethanol (TCE) (Sigma-Aldrich, Saint Louis, MO, USA, T54801-500G), which allows direct visualization of proteins under UV illumination without staining and does not interfere with downstream Western blotting. After electrophoretic separation, protein bands were imaged by UV activation of TCE using a ChemiDoc MP Imaging System (Bio-Rad, Hercules, CA, USA).

Proteins were transferred to a polyvinylidene fluoride (PVDF) membrane using the transblot transfer kit (Cat:1704274, Bio-Rad) at 25 V for 30 min using the Trans-blot Turbo transfer system (Bio-Rad, Hercules, CA, USA). The PVDF membranes were blocked for 30 min at RT in 5% nonfat dried milk in Tris Buffered Saline with 0.1% (TBST) or in EveryBlot Blocking Buffer (Bio-Rad, Hercules, CA, USA, 12010020) for 5–10 min at RT. Membranes were probed overnight at 4 °C with the following primary antibodies, diluted in blocking buffer as indicated: anti-L-plastin (1:500 in EveryBlot), anti-GFP antibody (1:60,000 in 5% non-fat milk), or Myo10 (1:3000 in 5% non-fat milk). After 5 washes (5 min each in TBST at RT), membranes were incubated for 50 min at RT in the dark with Starbright Blue 700 fluorescent secondary antibodies: goat anti-rabbit (1:3000 in 5% non-fat milk) (Bio-Rad, Hercules, CA, USA, 12004161) and goat anti-mouse (1:3000 in Bio-Rad Every Blot, 12010020) (Bio-Rad, 12004161), with 0.02% SDS. After secondary antibody incubation, membranes were washed 5 times (5 min each) in TBST and imaged with a Bio-Rad ChemiDoc MP imaging system. Band intensities were quantified using Image Lab Software version 6.1.0 build 7 Standard Edition (Bio-Rad) to assess protein expression levels between control and experimental groups.

### 2.6. Statistical Analyses

The Student *t*-test was used to analyze statistical significance for our Western blot experiments between independent treatment groups. This parametric test was applied after confirming that the data met assumptions of approximate normality and homogeneity of variances. Data are presented as mean ± standard errors (s.e.m).

The Mann-U Whitney test was used for the TNT counting experiments. The data were not normally distributed, prompting the use of a nonparametric approach to compare two independent groups. This rank-based test assessed whether the distributions of the groups differed, and calculations were performed using VassarStats (http://vassarstats.net/utest.html (accessed 20 March 2025)). For our results, two-tailed *p*-values with a *p* < 0.05 were considered statistically significant and represented by one star (*).

## 3. Results

### 3.1. L-Plastin Localizes to Stressed-Induced Tunneling Nanotubes and Proto-TNTs

**Figure 1:**
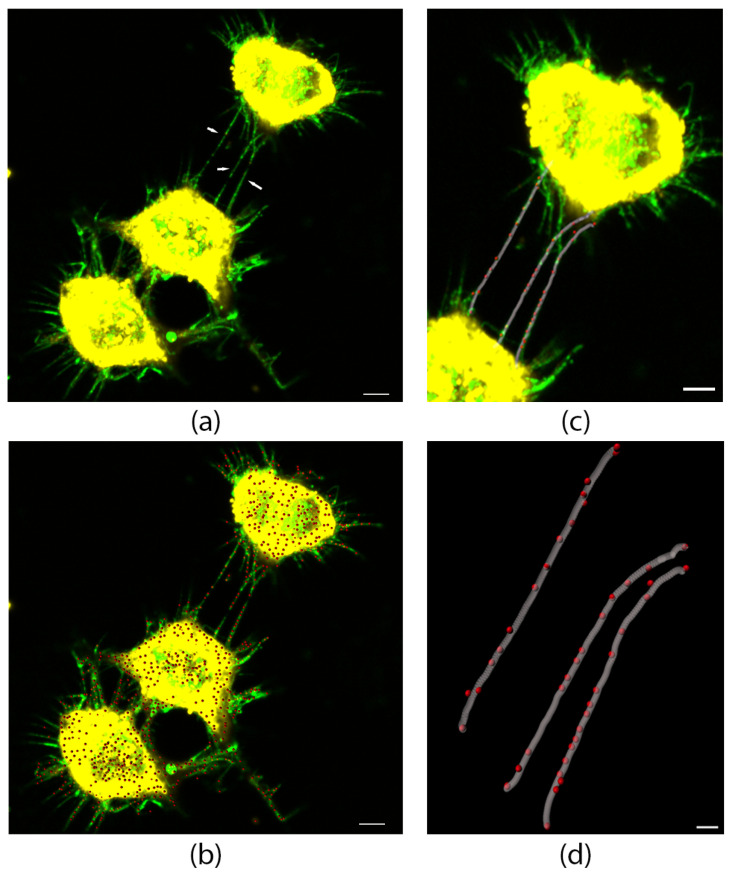
L-plastin localizes within TNTs and proto-TNTs in CAD cells treated with hydrogen peroxide. CAD cells were treated with 100 μM H_2_O_2_ for 5 min to increase the number of TNTs between cells, fixed and labeled with anti-L-plastin antibody (red) and the membrane dye WGA-green to identify cellular protrusions (green). (**a**) A confocal microscopy 3D representative image of CAD cells connected with TNTs (white arrows). (**b**) 3D reconstruction image using Imaris software, employing “spot rendering” to identify L-plastin localization (red) within TNTs and along proto-TNTs. (**c**) 3D reconstruction image using Imaris software, employing “spot rendering” plus “filament tracer” to enhance the visualization of L-plastin puncta (in red) within TNTs (gray). (**d**) Zoom in of the 3D reconstruction using filament tracer and spot rendering in (**c**). All scale bars are 2 μm in length.

To investigate the potential role of L-plastin in stressed-induced TNTs, we first examined its subcellular distribution in CAD cells, a well-established model for TNT formation under stress conditions [9,22,30], treated with H_2_O_2_. After H_2_O_2_ treatment, the cells were labeled with WGA-green, to delineate the plasma membrane and identify TNTs [22,30,47,48] and immunolabeled with anti-L-plastin antibody. Confocal microscopy images revealed the presence of punctated L-plastin signals along thin, hovering membrane tubes that connected distant cells—structures morphologically defined as TNTs (Figure 1a). Three-dimensional reconstruction using Imaris software, with spot rendering for L-plastin puncta and filament tracer for the TNT membrane backbone, confirmed that L-plastin puncta were embedded within the TNT shaft (Figure 1b–d).

These puncta were distributed throughout the length of the tubes, with no preferential accumulation at the base, midpoint, or tip. In addition to TNTs, L-plastin puncta were also visible in numerous filopodia-like protrusions or what could be proto-TNTs extending from the cell surface, a localization that is consistent with its known role as an actin-bundling protein in dynamic membrane extensions. These initial observations supported the hypothesis that L-plastin is a structural component of TNTs and potentially contributes to their formation or stability.

### 3.2. Overexpression of L-Plastin Increases TNT Formation

**Figure 2:**
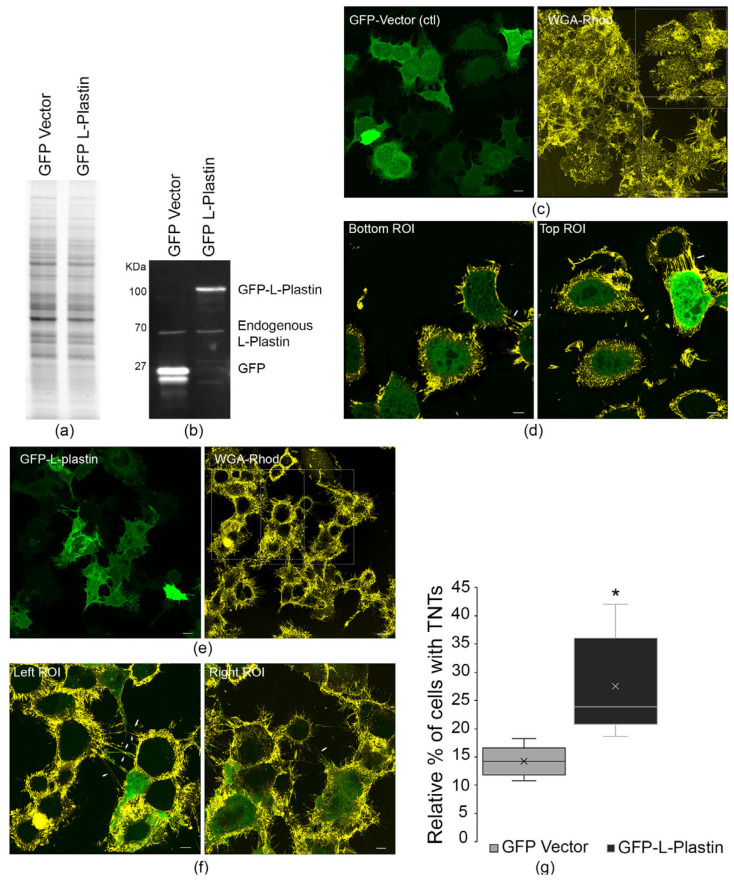
L-plastin upregulation increases the number of cells connected by TNTs. CAD cells were transiently transfected with either GFP-vector as a control or GFP-L-plastin for 24 h and lysed. (**a**) Representative stain free gel is shown as a loading control. Stain free gels were used to quantify the total protein loaded in each lane. (**b**) Representative fluorescent Western blot shows upregulation of L-plastin protein levels in GFP-L-plastin transfected cells compared to cells transfected with GFP-vector control. Parallel experiments were performed along with the Western blots. CAD cells transfected with GFP-vector or GFP-L-plastin were imaged and Z-stacks of random fields were acquired. (**c**) Representative 3D image of GFP transfected cells is shown for the green channel (GFP) and red channel (WGA-Rhod). (**d**) Zoom in of ROIs (dashed rectangles) outlined in (**c**), where TNTs (white arrowhead) are present. (**e**) Representative 3D image of GFP-L-plastin transfected cells is shown for the green channel (GFP) and red channel (WGA-Rhod). (**f**) Zoom in of ROIs (dashed rectangles) outlined in (**e**), where TNTs are present (white arrowhead). All the experiments were acquired and analyzed under blinded conditions to minimize observer bias. Scale bars for (**c**,**e**) = 10 μm and 5 μm for (**d**,**f**). (**g**) Overexpression of GFP-L-plastin resulted in an increase in the number of cells connected by TNTs. The average percentage of cells connected by TNTs was 27.2% (±4.1%) for GFP-L-plastin compared to 14.2% (±1.2%) for GFP-vector. Data are the average of 5 independent experiments (*n* = 588 GFP-vector and *n* = 582 for GFP-L-plastin). Graph show means (±s.e.m; *p* = 0.0121). * *p* < 0.05.

We next tested whether increasing L-plastin expression levels would affect TNT formation. CAD cells were transfected with GFP-vector, as a control, or GFP-L-plastin constructs, and protein overexpressions were analyzed by fluorescent Western blotting (Figure 2a,b). Stain-free total protein normalization and quantification showed robust upregulation of both transfected GFP-L-plastin and endogenous L-plastin in the GFP-L-plastin transfected cells compared to GFP-vector control.

Next, we quantified the number of cells connected by TNTs. To do this, we acquired Z-stack images from random fields of CAD cells transfected with either GFP-vector or GFP-L-plastin and labeled the cells with WGA-Rhodamine to identify TNTs (Figure 2c,e). As can be seen in Figure 2c (WGA-Rhod), CAD cells have numerous attached filopodia as well as dorsal filopodia. To properly identify and distinguish between filopodia and TNTs, the bottom Z-stacks, representing attached filopodia were discarded, and TNTs were identified as any structures connecting two cells and not attached to the substratum. Representative Z-stack images for one field of view are shown in Figure S1 for both GFP-vector transfected cells and GFP-L-plastin transfected cells (Figure S1a,b). For the control GFP-transfected cells in Figure S1a, three transfected cells out of 18 cells were connected with TNTs (16.67%). In Figure S1b, we observed 18 GFP-L-plastin transfected cells and seven were connected with TNTs (38.89%). To quantify the effects of L-plastin over-expression, five independent experiments were obtained, and a total of 588 GFP-vector transfected cells were counted and compared to 582 GFP-L-plastin transfected cells. To avoid observer biases, the experiments were conducted under blinded conditions, for the entirety of the experiments (i.e., image acquisition and TNT counting). As shown in Figure 2g, L-plastin overexpression significantly increased the percentage of TNT-connected cells (27.2 ± 4.1% versus 14.2 ± 1.2% for GFP-vector transfected cells). These data indicate that higher L-plastin availability is necessary for maintaining normal TNT abundance under these conditions and promotes TNT formation and/or stability.

### 3.3. Knockdown of L-Plastin Reduces TNT Numbers

**Figure 3:**
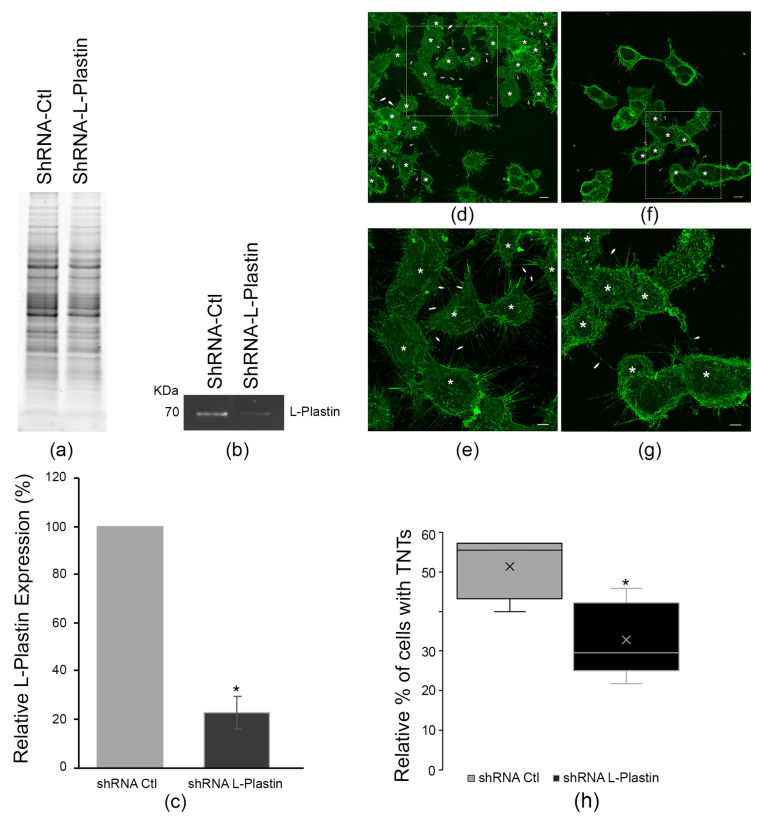
L-plastin downregulation decreases the number of cells connected by TNTs. (**a**) Representative stain free gel is shown as a loading control. (**b**) Representative fluorescent Western blot demonstrating successful downregulation of L-plastin protein levels in shRNA-L-plastin cells compared to the shRNA-Control. (**c**) Quantification of L-plastin downregulation from Western blots shows a 77.2% downregulation in L-plastin expression (22.8 ± 6.48) compared to control cells. Data are the average of 5 independent experiments. Graph show means (±s.e.m; *p* = 0.0121). (**d**) Top Z-stack representative 3D image of control shRNA transduced cells and (**e**) Zoom in of ROI outlined in (dashed rectangles) (**d**). (**f**) Representative 3D image of shRNA L-plastin transduced cells and (**g**) Zoom in of ROI outlined in (dashed rectangles) (**f**). TNTs are highlighted with white arrowheads and cells connected by TNTs are identified with a white star (*). Scale bars for (**d**,**f**) = 10 μm and 5 μm for (**e**,**g**). Scale bars = 5 μm. (**h**) Down regulation of L-plastin resulted in a significantly lower percentage of cells connected by TNT (32.7 ± 4.7%) compared to the shRNA-control cells (51.2 ± 3.8%). The quantification of cells connected by TNTs came from 5 blinded independent experiments (*n* = 520 for shRNA Ctl and *n* = 508 for shRNA L-plastin). Graph show means (±s.e.m; *p* = 0.0214). * *p* < 0.05.

To assess whether L-plastin is required for TNT formation and function, we generated stable L-plastin knockdown lines using shRNA lentiviral particles. Fluorescent Western blotting demonstrated efficient depletion of L-plastin protein (77.2% reduction versus shRNA-control; Figure 3a–c).

In parallel, TNT quantification experiments were conducted. In order to maintain stable transduced clones, the cells were grown in media supplemented with puromycin. Representative images of the shRNA control and shRNA L-plastin cells are shown in Figure S2 and Figure 3d,f, respectively. Compared to CAD cells grown without antibiotics (Figure 2), the transduced cells grown in puromycin were more stressed as seen by the increased length in the adhering filopodia (Figure S2). This was expected since puromycin is a known stress inducer in cells under selection [49]. Similar to Figure 2, to identify TNTs, cells were identified by going through the Z-stacks. The bottom Z-stacks corresponding to adhering filopodia (Figure S2b,e) were discarded and structures connecting cells, above the substratum in the middle and top Z-stacks, were counted (Figure S2c,f). In the representative image shown in Figure S2 for the shRNA control, we had 42 cells with 21 cells connected with TNTs as denoted with the white stars and 24 cells of the shRNA L-plastin with seven connected with TNTs. Thus, in these examples, the shRNA control had 50% of cells connected with TNTs and ~29% of shRNA L-plastin cells were connected with TNTs. Enlarged images of cells connected by TNTs from Figure S2 are shown in Figure 3d,e. To quantify the effects of L-plastin down regulation, five independent blinded experiments were performed, and a total of 502 shRNA control cells were counted vs. 508 shRNA L-plastin cells. L-plastin knockdown significantly decreased TNT-connected cells (32.7 ± 4.7% versus 51.2 ± 3.8% in shRNA-control). The overall increase in the number of cells connected with TNTs in Figure 3 compared to Figure 2 is likely due to the presence of puromycin in the media, stressing the cells and increasing the number of stress-induced TNTs. However, it is interesting to note that once again, in these stress-induced cells, L-plastin is required to maintain stable TNTs. Together with the overexpression data, these results establish L-plastin as both sufficient and required for normal TNT formation.

### 3.4. L-Plastin Levels Do Not Reciprocally Regulate Myosin-X Expression

**Figure 4:**
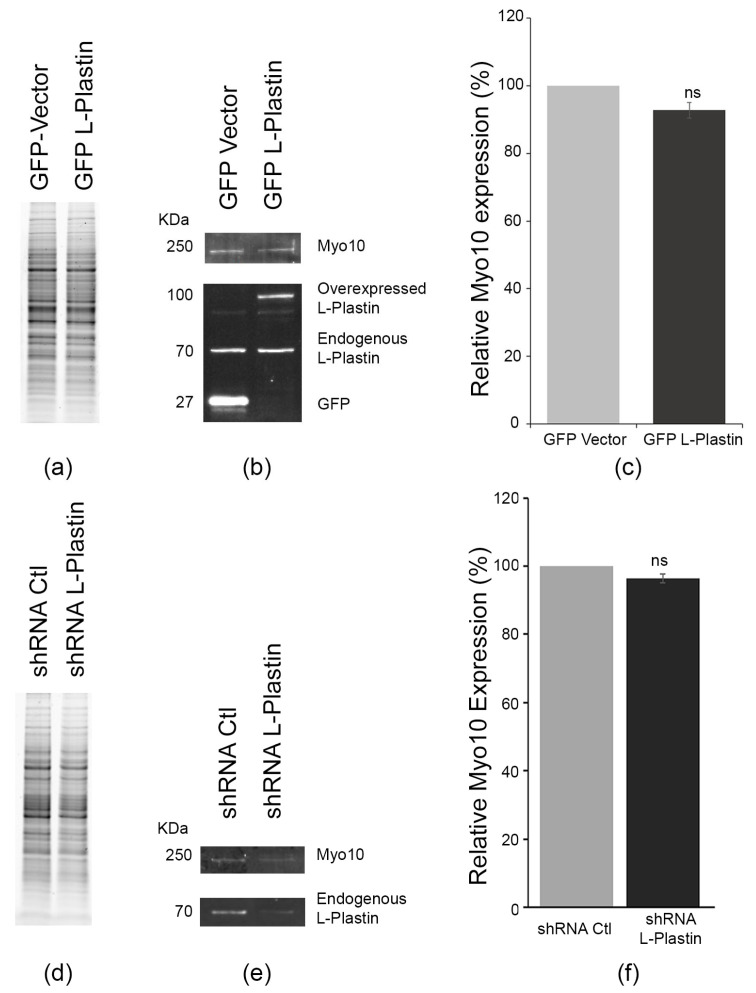
L-plastin expression levels dos not affect Myo10 expression. (**a**) Representative stain free gel is shown as a loading control. (**b**) Representative fluorescent Western blot showing up-regulation of L-plastin expression and its effect on Myo10 protein levels. (**c**) Quantitative analysis showing no statistical significant changes (ns) in Myo10 expression (92.8% ± 2.3%; *p* = 0.9203) upon L-plastin upregulation. (**d**,**e**) Stain free gel and Western blot from Figure 3a,b showing successful down-regulation of L-plastin (~70 KDa) in ShRNA L-plastin cells. To see its effect on Myo10 expression, the top part of the blot was probed with Myo10 antibody to determine the effect of L-plastin down regulation on Myo10 protein levels (~250 KDa). (**f**) Quantification of relative levels of Myo 10 expression shows no significant changes (96.4%; (ns) with *p* = 0.1443) compared to control cells. Data are the average of 5 independent experiments, and the graph shows means (±s.e.m).

Given the established role of Myo10 in TNT formation, we asked whether differential L-plastin levels affect Myo10 expression levels. Myo10 protein levels remained unchanged in CAD cells overexpressing GFP-L-plastin compared to GFP-vector controls (92.8 ± 2.3% of control; *p* = 0.9203, not significant; Figure 4a–c). Similarly, shRNA-mediated L-plastin knockdown had no significant effect on Myo10 expression (96.4% of shRNA-control levels; *p* = 0.1443, not significant; Figure 4d–f). These data demonstrate unidirectional regulation, where L-plastin influences TNT formation without modulating Myo10 expression.

### 3.5. Myosin-X Controls L-Plastin Protein Abundance and TNT Formation

**Figure 5:**
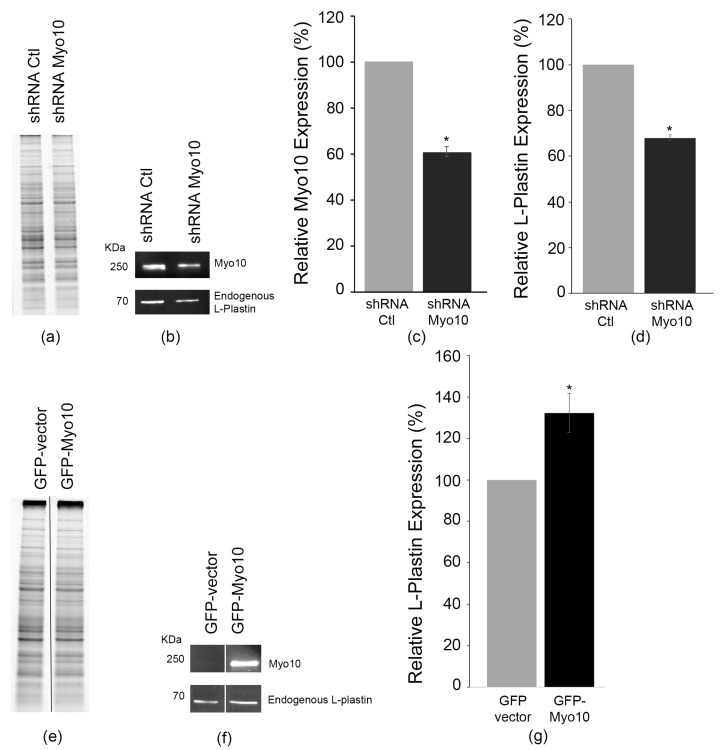
Myo10 expression levels affect L-plastin expression. (**a**) Representative stain free gel is shown as a loading control. (**b**) Representative fluorescent Western blot showing that down-regulation of Myo10 expression (61.0% ± 2.4% with *p* = 0.0121) using shRNA lentiviral particles (**c**) results in the reduction of L-plastin expression levels to 68% (±2.8%; *p*-value: 0.0121). Data are the average of 5 independent experiments. (**d**) Conversely, we looked at the effects of up-regulation of Myo10 using transient GFP-Myo10 transfection. (**e**) Representative stain free gel is shown as a loading control. To avoid that the very bright GFP signal affects the endogenous Myo10 signal, the GFP-vector experiments were loaded first, followed by the GFP-Myo10 transfected cells on the same gels. Here a representative experiment is shown, where the control lane (GFP-vector) and its matching experimental lane (GFP-Myo10) from the same gel were juxtaposed as shown by the black separation lane. The entire gel is shown in Figure S3; lanes 1 (**e**) and 4 (**f**) were used as a representative image here. (**f**) Representative fluorescent Western blot of the same samples as (**e**) are shown and it demonstrates that up-regulation of GFP-Myo10 expression results in the upregulation of endogenous L-plastin expression. (**g**) Quantitative analysis shows a 132.3.3% (±9.4%; *p* = 0.0121) increase in L-plastin in cells over-expressing GFP-Myo10 compared to the control GFP expressing cells. Data are the average of 5 independent experiments. * *p* < 0.05.

We next examined whether Myo10 regulates L-plastin expression levels. We previously showed that full length Myo10 is necessary for TNT formation and that the number of TNTs increase in GFP-Myo10 transfected cells [22]. Conversely, we found that down-regulation of Myo10 resulted in a decrease in the number of cells connected by TNTs [22]. Thus, we looked whether stable shRNA knockdown of Myo10 would affect L-plastin expression levels. Here we found that CAD cells with ~39% reduction in Myo10 protein expression levels resulted in decreased endogenous L-plastin levels by 30.8 ± 2.4% versus shRNA-control (Figure 5a–d).

Conversely, we looked at whether overexpression of GFP-Myo10 would affect L-plastin levels. Because of the very bright GFP-Myo10 signals, we did not run the GFP transfected control experiments next to the GFP-Myo10 transfected samples. Instead, we ran the control experiments first and separated them from the GFP-Myo10 samples with a protein ladder (Figure S3). A representative experiment shows lane 1 and 4 from Figure S3 called Figure 5e,f. As can be seen in Figure 5e–g, transient GFP-Myo10 overexpression resulted in a 132.4% (±9.4%) increase in endogenous L-plastin levels.

Together, these results position Myo10 upstream of L-plastin in a regulatory hierarchy controlling TNT formation and reveal an unexpected role for the TNT initiation molecular motor in sustaining the pool of a key structural bundler like L-plastin, necessary for TNT formation and stability.

### 3.6. Myosin-X and L-Plastin Co-Localize in TNTs and Proto-TNTs

**Figure 6:**
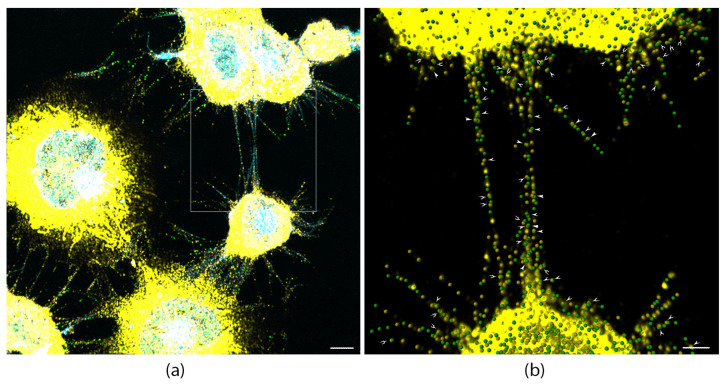
Myo10 and L-plastin co-localize in TNTs and proto-TNTs. CAD cells were treated with H_2_O_2_ in order to increase the number of cells connected by TNTs, then labeled with anti-L-plastin (yellow) and anti-Myo10 antibodies (green), WGA-350 (Blue). (**a**) A confocal microscopy representative image of CAD cells connected with TNTs is shown. 3D reconstruction using Imaris software, employing “spot rendering” was used to highlight endogenous L-plastin (yellow punctae) and endogenous Myo10 (green punctae), and WGA-350 was used to delineate TNTs. Both proteins are found throughout TNTs and proto-TNTs. Scale bar = 5 μm. (**b**) Zoom in of (**a**) shows that endogenous L-plastin punctates (yellow) colocolize with Myo10 punctates (green) within these structures (white arrows). Scale bar = 2 μm.

Finally, dual-immunofluorescence imaging showed co-localization of Myo10 and L-plastin along TNT shafts and proto-TNTs (Figure 6a,b).

High-magnification zooms and 3D Imaris spot rendering demonstrated that both proteins decorate the same TNT shafts, with visually evident overlap (white arrows in Figure 6b) and were distributed throughout these structures, consistent with cooperative function in protrusion elongation (Myo10) and bundling/stability (L-plastin).

Taken together, these data establish a unidirectional Myo10–L-plastin axis that regulates stress-induced TNT formation: Myo10 acts as a protrusive motor and upstream organizer that controls both acute recruitment/activation and overall protein expression levels of L-plastin, while L-plastin stabilizes the parallel actin bundles that form the TNT core.

## 4. Discussion

In this study, we identified Myo10 as an upstream regulator of L-plastin during the formation and maintenance of tunneling nanotubes under cellular stress. Using gain- and loss-of-function approaches in CAD cells, we showed that Myo10 levels and L-plastin protein levels change in parallel—Myo10 overexpression increases L-plastin, whereas Myo10 knockdown decreases it—while L-plastin manipulation has no reciprocal effect on Myo10 (Figure 4 and Figure 5). In contrast, altering L-plastin levels alone is sufficient to modulate TNT formation, with L-plastin overexpression increasing the number of cells connected by TNTs and L-plastin depletion decreasing the proportion of TNT-connected cells (Figure 2 and Figure 3). Both proteins extensively co-localize along TNT shafts and proto-TNTs (Figure 6), and depletion of either reduces the formation of TNTs, underscoring that both are required for maintaining normal TNT networks. Together with our previous demonstration that Myo10 controls TNT formation in CAD cells [22], these findings define a unidirectional Myo10-L-plastin axis and position Myo10 as an upstream organizer that governs both the availability and deployment of the downstream actin-bundling factor.

The requirement for Myo10 in TNT formation has been firmly established in CAD cells and other systems [17,19,22,30]. Our results agree with this foundational role and help explain how Myo10 interfaces with downstream cytoskeletal regulators to convert protrusive activity into stable actin-based nanotubes. Work in filopodia and related protrusions has identified the Myo10–integrin–PI3K positive-feedback loops that locally enrich PIP_3_ and kinase activity [25,26,27,28]. Such mechanisms provide a plausible context for the microenvironment in which L-plastin becomes activated. In particular, PI3K-dependent PIP_2_ depletion and phosphorylation of L-plastin at Ser5 by SGK have been shown to enhance its F-actin affinity, increase bundling activity, and relieve Ca^2+^-mediated inhibition [37,38,39]. These prior mechanistic insights are consistent with a model in which Myo10-dependent PI3K signaling creates PIP_3_-enriched membrane patches at TNT tips that both recruit Myo10 and favor L-plastin activation, allowing TNT shafts to be stabilized by tightly bundled F-actin.

Proto-TNTs are morphologically filopodia-like, and TNT biogenesis is therefore expected to share many of the same upstream components that also drive filopodia initiation and elongation, including Ca^2+^/calmodulin-dependent activation of Myo10 and Myo10-associated PI3K signaling. Importantly, however, TNTs are not simply filopodia: they mature, most likely due to stress conditions, into non-adherent, free-spanning intercellular bridges that must persist long enough to function as conduits for cell–cell transfer. Moreover, the transition from filopodia-like proto-TNTs to mature TNTs imposes discrete mechanical and organizational requirements compared to substrate-attached filopodia, including the stabilization of long, parallel actin bundles that resist membrane tension and shear while remaining suspended above the substratum. Our data support a division of labor in which Myo10 provides the upstream protrusive framework (a feature shared with filopodia and other actin-based protrusions). In contrast, L-plastin functions downstream as a key actin-bundling/stabilizing effector that promotes TNT shaft integrity and network maintenance during cellular stress. Thus, the Myo10/L-plastin hierarchy we define is best viewed as a TNT ‘initiation-to-stabilization’ pathway: it uses the same Myo10-dependent mechanisms that initiate filopodia-like protrusions but directs the structure toward a mature TNT outcome by recruiting L-plastin to stabilize the actin bundles against the mechanical stresses encountered in suspended, intercellular TNTs.

Our data also reveal a longer-timescale relationship in which Myo10 levels influence total L-plastin protein levels (Figure 4 and Figure 5). Although the underlying mechanism remains to be clarified, this observation suggests that Myo10 may contribute to sustaining the pool of L-plastin available for TNT assembly during prolonged stress, in addition to its role in protrusion initiation. Recent reports in breast preneoplastic models describe rapid L-plastin upregulation in response to TNT-mediated signaling [46], supporting the broader concept that L-plastin levels can be adaptively modulated during periods of high TNT activity. In that system, elevated L-plastin correlated with increased protrusion formation and invasive behavior—paralleling the enhanced TNT abundance we observed upon L-plastin overexpression (Figure 2) and consistent with its established roles in migration and metastasis [50,51,52]. Together, these findings raise the possibility that Myo10-dependent signaling, downstream growth factor pathways, and TNT-mediated communication converge on L-plastin as a central actin-bundling effector.

The strictly unidirectional nature of the axis—Myo10 regulates L-plastin, but not vice versa (Figure 4 and Figure 5)—helps reconcile disparate observations in the TNT literature. Myo10 is essential for protrusion initiation and elongation [21,22,23,24], whereas L-plastin provides structural reinforcement once parallel actin bundles are assembled [50]. Our results integrate these functions: Myo10 establishes the protrusive framework and associated signaling environment that recruit and activate L-plastin along the nanotube shaft. In Myo10-downregulated cells, this environment is not established, L-plastin levels decline, and TNTs fail to form or remain unstable, a phenotype consistent with prior reports of Myo10 loss-of-function [22]. Conversely, elevating L-plastin in the presence of normal endogenous levels of Myo10 increases TNT abundance, highlighting its role as a key structural effector rather than a primary initiator.

These findings have broad implications. Physiologically, the Myo10–L-plastin axis provides a mechanism through which transient Ca^2+^-dependent stress signals can be converted into stable intercellular conduits for mitochondrial or signaling transfer [5,6,7,8]. In pathological settings, dysregulation of the same pathways—via hyperactive PI3K/AKT signaling, elevated Myo10 expression, or chronic L-plastin upregulation—could promote excessive TNT formation, supporting tumor–stroma communication, metabolic coupling, and therapy resistance [9,10,11,12,13,14,15,16,17,30]. Components of the Myo10–integrin–PI3K pathway, and potentially L-plastin itself, may therefore represent selective targets for disrupting TNT networks in cancer without broadly compromising other actin-based structures.

Several questions remain. The identity of transcriptional or post-transcriptional mediators that couple Myo10 perturbation to changes in L-plastin protein levels is unknown and warrants investigation. Likewise, the role of calcium-dependent proteases such as calpain—which can process Myo10 under sustained stress [53]—in tuning the dynamics or lifetime of Myo10- and L-plastin-positive TNTs remains to be tested. Finally, extending these findings to other cell types, primary cells, and in vivo models will be important for determining the generality of this regulatory hierarchy across tissues and disease contexts.

## 5. Conclusions

In conclusion, we define a hierarchical Myo10–L-plastin axis that links stress-induced Ca^2+^ signaling and Myo10-dependent protrusion initiation to L-plastin-mediated actin-bundle stabilization. This framework connects TNT initiation, maintenance, and stress adaptation, and provides new insights into the molecular logic underlying intercellular communication in both homeostasis and disease.

## Data Availability

The original contributions presented in this study are included in the article/Supplementary Material. Further inquiries can be directed to contact K. Gousset (kgousset@mail.fresnostate.edu).

## Supplementary Materials

The following supporting information can be downloaded at: https://www.mdpi.com/article/10.3390/cells15030224/s1, Figure S1: L-plastin Upregulation Increases the number of cells connected by TNTs. CAD cells transfected with GFP-vector or GFP-L-plastin were imaged and Z-stacks of random fields were acquired. To identify TNTs, Z-stacks are analyzed going from the bottom of the Z-stack (i.e. substratum) to the top of the cells. (**a**) Representative 3D images from the Z-stacks acquired for Figure 2c are shown for the control GFP transfected cells. (**b**) Representative 3D images from the Z-stacks acquired for Figure 2e are shown for the GFP-L-plastin transfected cells. TNTs are highlighted with white arrowheads and transfected cells connected by TNTs are noted with white stars (*). All the experiments were acquired and analyzed under blinded conditions to minimize observer bias. Scale bars = 10 um. Figure S2: L-plastin downregulation decreases the number of cells connected by TNTs. (**a**) Representative 3D image of control shRNA transduced cells. (**b**) Bottom Z-stacks from (**a**) representing adhering filopodia. (**c**) Top Z-stacks from (**a**) to identify TNTs (white arrowheads). Cells connected with TNTs are highlighted with a white star (*). (**d**) Representative 3D image of shRNA L-plastin transduced cells. (**e**) Bottom Z-stacks from (**d**) representing adhering filopodia. (**f**) Top Z-stacks from (**d**) to identify TNTs (white arrowheads); Figure S3: Myo10 expression levels affect L-plastin expression. Three independent experiments were loaded on the gel. The GFP transfected control samples were ran first, followed by the samples transfected with GFP-Myo10 because of the very bright signals from GFP-Myo10 transfection. (**a**) The stain free gel is shown as a loading control. (**b**) Representative fluorescent Western blot showing the levels of Myo10 and endogenous levels of L-plastin in these experiments. Lane 1 (GFP-vector expt#1) and lane 4 (GFP-Myo10 expt#1) are the representative lanes juxtaposed in Figure 5e,f. Cells connected with TNTs are highlighted with a white star (*). Scale bars =10 μm.

## Abbreviations

The following abbreviations are used in this manuscript:

TNT	Tunneling nanotube
CAD	Cath. a-differentiated neuronal cells
Myo10	Myosin-X
LCP1	L-plastin
Proto-TNT	Filopodia-like TNT precursor
HIV-1	Human Immunodeficiency Virus, type 1
ROS	Reactive Oxygen Species
mtROS	Mitochondrial Reactive Oxygen Species
Ca^2+^	Calcium
PIP3	Phosphatidylinositol 3,4,5-trisphosphate
PIP2	Phosphatidylinositol 4,5-bisphosphate
PH	Pleckstrin homology
PI3K	Phosphoinositide 3-Kinase
SGK	Serum/Glucocorticoid-regulated Kinase
LCM	Laser Capture Microsdissection
WGA	Wheat Germ Agglutinin
PFA	Paraformaldehyde
DTBP	Dithiobispropionimidate
shRNA	Short hairpin RNAPBS
PBS	Phosphate-Buffered Saline
TCE	2,2,2-Trichloroethanol
PVDF	Polyvinylidene Fluoride
TBST	Tris Buffered Saline with 0.1% Tween
RT	Room temperature
Standard Error	S.E.
control	ctl
micrometer	um
ROI	Region of Interest

## References

[1] Rustom A., Saffrich R., Markovic I., Walther P., Gerdes H.-H. (2004). Nanotubular Highways for Intercellular Organelle Transport. Science.

[2] Abounit S., Zurzolo C. (2012). Wiring through Tunneling Nanotubes--from Electrical Signals to Organelle Transfer. J. Cell Sci..

[3] Sisakhtnezhad S., Khosravi L. (2015). Emerging Physiological and Pathological Implications of Tunneling Nanotubes Formation between Cells. Eur. J. Cell Biol..

[4] Gerdes H.-H., Rustom A., Wang X. (2013). Tunneling Nanotubes, an Emerging Intercellular Communication Route in Development. Mech. Dev..

[5] Wang X., Gerdes H.-H. (2015). Transfer of Mitochondria via Tunneling Nanotubes Rescues Apoptotic PC12 Cells. Cell Death Differ..

[6] Islam M.N., Das S.R., Emin M.T., Wei M., Sun L., Westphalen K., Rowlands D.J., Quadri S.K., Bhattacharya S., Bhattacharya J. (2012). Mitochondrial Transfer from Bone-Marrow-Derived Stromal Cells to Pulmonary Alveoli Protects against Acute Lung Injury. Nat. Med..

[7] Onfelt B., Nedvetzki S., Benninger R.K.P., Purbhoo M.A., Sowinski S., Hume A.N., Seabra M.C., Neil M.A.A., French P.M.W., Davis D.M. (2006). Structurally Distinct Membrane Nanotubes between Human Macrophages Support Long-Distance Vesicular Traffic or Surfing of Bacteria. J. Immunol..

[8] Spees J.L., Olson S.D., Whitney M.J., Prockop D.J. (2006). Mitochondrial Transfer between Cells Can Rescue Aerobic Respiration. Proc. Natl. Acad. Sci. USA.

[9] Gousset K., Schiff E., Langevin C., Marijanovic Z., Caputo A., Browman D.T., Chenouard N., de Chaumont F., Martino A., Enninga J. (2009). Prions Hijack Tunnelling Nanotubes for Intercellular Spread. Nat. Cell Biol..

[10] Eugenin E.A., Gaskill P.J., Berman J.W. (2009). Tunneling Nanotubes (TNT) Are Induced by HIV-Infection of Macrophages: A Potential Mechanism for Intercellular HIV Trafficking. Cell. Immunol..

[11] Victoria G.S., Zurzolo C. (2015). Trafficking and Degradation Pathways in Pathogenic Conversion of Prions and Prion-like Proteins in Neurodegenerative Diseases. Virus Res..

[12] Desir S., Wong P., Turbyville T., Chen D., Shetty M., Clark C., Zhai E., Romin Y., Manova-Todorova K., Starr T.K. (2019). Intercellular Transfer of Oncogenic KRAS via Tunneling Nanotubes Introduces Intracellular Mutational Heterogeneity in Colon Cancer Cells. Cancers.

[13] Thayanithy V., Dickson E.L., Steer C., Subramanian S., Lou E. (2014). Tumor-Stromal Cross Talk: Direct Cell-to-Cell Transfer of Oncogenic microRNAs via Tunneling Nanotubes. Transl. Res. J. Lab. Clin. Med..

[14] Pasquier J., Guerrouahen B.S., Al Thawadi H., Ghiabi P., Maleki M., Abu-Kaoud N., Jacob A., Mirshahi M., Galas L., Rafii S. (2013). Preferential Transfer of Mitochondria from Endothelial to Cancer Cells through Tunneling Nanotubes Modulates Chemoresistance. J. Transl. Med..

[15] Kretschmer A., Zhang F., Somasekharan S.P., Tse C., Leachman L., Gleave A., Li B., Asmaro I., Huang T., Kotula L. (2019). Stress-Induced Tunneling Nanotubes Support Treatment Adaptation in Prostate Cancer. Sci. Rep..

[16] Valdebenito S., Malik S., Luu R., Loudig O., Mitchell M., Okafo G., Bhat K., Prideaux B., Eugenin E.A. (2021). Tunneling Nanotubes, TNT, Communicate Glioblastoma with Surrounding Non-Tumor Astrocytes to Adapt Them to Hypoxic and Metabolic Tumor Conditions. Sci. Rep..

[17] Tiwari V., Koganti R., Russell G., Sharma A., Shukla D. (2021). Role of Tunneling Nanotubes in Viral Infection, Neurodegenerative Disease, and Cancer. Front. Immunol..

[18] Lin X., Wang W., Chang X., Chen C., Guo Z., Yu G., Shao W., Wu S., Zhang Q., Zheng F. (2024). ROS/mtROS Promotes TNTs Formation via the PI3K/AKT/mTOR Pathway to Protect against Mitochondrial Damages in Glial Cells Induced by Engineered Nanomaterials. Part. Fibre Toxicol..

[19] Vargas J.Y., Loria F., Wu Y.-J., Córdova G., Nonaka T., Bellow S., Syan S., Hasegawa M., van Woerden G.M., Trollet C. (2019). The Wnt/Ca^2+^ Pathway Is Involved in Interneuronal Communication Mediated by Tunneling Nanotubes. EMBO J..

[20] Berg J.S., Cheney R.E. (2002). Myosin-X Is an Unconventional Myosin That Undergoes Intrafilopodial Motility. Nat. Cell Biol..

[21] Tokuo H., Ikebe M. (2004). Myosin X Transports Mena/VASP to the Tip of Filopodia. Biochem. Biophys. Res. Commun..

[22] Gousset K., Marzo L., Commere P.-H., Zurzolo C. (2013). Myo10 Is a Key Regulator of TNT Formation in Neuronal Cells. J. Cell Sci..

[23] Kerber M.L., Cheney R.E. (2011). Myosin-X: A MyTH-FERM Myosin at the Tips of Filopodia. J. Cell Sci..

[24] Zhang H., Berg J.S., Li Z., Wang Y., Lång P., Sousa A.D., Bhaskar A., Cheney R.E., Strömblad S. (2004). Myosin-X Provides a Motor-Based Link between Integrins and the Cytoskeleton. Nat. Cell Biol..

[25] Jacquemet G., Hamidi H., Ivaska J. (2015). Filopodia in Cell Adhesion, 3D Migration and Cancer Cell Invasion. Curr. Opin. Cell Biol..

[26] Miihkinen M., Grönloh M.L.B., Popović A., Vihinen H., Jokitalo E., Goult B.T., Ivaska J., Jacquemet G. (2021). Myosin-X and Talin Modulate Integrin Activity at Filopodia Tips. Cell Rep..

[27] Anthis N.J., Wegener K.L., Ye F., Kim C., Goult B.T., Lowe E.D., Vakonakis I., Bate N., Critchley D.R., Ginsberg M.H. (2009). The Structure of an Integrin/Talin Complex Reveals the Basis of inside-out Signal Transduction. EMBO J..

[28] Jacquemet G., Baghirov H., Georgiadou M., Sihto H., Peuhu E., Cettour-Janet P., He T., Perälä M., Kronqvist P., Joensuu H. (2016). L-Type Calcium Channels Regulate Filopodia Stability and Cancer Cell Invasion Downstream of Integrin Signalling. Nat. Commun..

[29] Tasca A., Astleford K., Lederman A., Jensen E.D., Lee B.S., Gopalakrishnan R., Mansky K.C. (2017). Regulation of Osteoclast Differentiation by Myosin X. Sci. Rep..

[30] Uhl J., Gujarathi S., Waheed A.A., Gordon A., Freed E.O., Gousset K. (2019). Myosin-X Is Essential to the Intercellular Spread of HIV-1 Nef through Tunneling Nanotubes. J. Cell Commun. Signal..

[31] Vignjevic D., Kojima S., Aratyn Y., Danciu O., Svitkina T., Borisy G.G. (2006). Role of Fascin in Filopodial Protrusion. J. Cell Biol..

[32] Henderson J.M., Ljubojevic N., Belian S., Chaze T., Castaneda D., Battistella A., Giai Gianetto Q., Matondo M., Descroix S., Bassereau P. (2023). Tunnelling Nanotube Formation Is Driven by Eps8/IRSp53-Dependent Linear Actin Polymerization. EMBO J..

[33] Delanote V., Vandekerckhove J., Gettemans J. (2005). Plastins: Versatile Modulators of Actin Organization in (Patho)Physiological Cellular Processes. Acta Pharmacol. Sin..

[34] Morley S.C. (2012). The Actin-Bundling Protein L-Plastin: A Critical Regulator of Immune Cell Function. Int. J. Cell Biol..

[35] Schaffner-Reckinger E., Machado R.A.C. (2020). The Actin-Bundling Protein L-Plastin-A Double-Edged Sword: Beneficial for the Immune Response, Maleficent in Cancer. Int. Rev. Cell Mol. Biol..

[36] Shinomiya H. (2012). Plastin Family of Actin-Bundling Proteins: Its Functions in Leukocytes, Neurons, Intestines, and Cancer. Int. J. Cell Biol..

[37] Janji B., Giganti A., De Corte V., Catillon M., Bruyneel E., Lentz D., Plastino J., Gettemans J., Friederich E. (2006). Phosphorylation on Ser5 Increases the F-Actin-Binding Activity of L-Plastin and Promotes Its Targeting to Sites of Actin Assembly in Cells. J. Cell Sci..

[38] Machado R.A.C., Stojevski D., De Landtsheer S., Lucarelli P., Baron A., Sauter T., Schaffner-Reckinger E. (2021). L-Plastin Ser5 Phosphorylation Is Modulated by the PI3K/SGK Pathway and Promotes Breast Cancer Cell Invasiveness. Cell Commun. Signal. CCS.

[39] Jones S.L., Wang J., Turck C.W., Brown E.J. (1998). A Role for the Actin-Bundling Protein L-Plastin in the Regulation of Leukocyte Integrin Function. Proc. Natl. Acad. Sci. USA.

[40] Ishida H., Jensen K.V., Woodman A.G., Hyndman M.E., Vogel H.J. (2017). The Calcium-Dependent Switch Helix of L-Plastin Regulates Actin Bundling. Sci. Rep..

[41] Babich A., Burkhardt J.K. (2013). Coordinate Control of Cytoskeletal Remodeling and Calcium Mobilization during T-Cell Activation. Immunol. Rev..

[42] Al Tanoury Z., Schaffner-Reckinger E., Halavatyi A., Hoffmann C., Moes M., Hadzic E., Catillon M., Yatskou M., Friederich E. (2010). Quantitative Kinetic Study of the Actin-Bundling Protein L-Plastin and of Its Impact on Actin Turn-Over. PLoS ONE.

[43] Gousset K., Gordon A., Kumar Kannan S., Tovar J. (2019). A Novel Microproteomic Approach Using Laser Capture Microdissection to Study Cellular Protrusions. Int. J. Mol. Sci..

[44] Gordon A., Kannan S.K., Gousset K. (2018). A Novel Cell Fixation Method That Greatly Enhances Protein Identification in Microproteomic Studies Using Laser Capture Microdissection and Mass Spectrometry. Proteomics.

[45] Gordon A., Gousset K. (2021). Utilization of Laser Capture Microdissection Coupled to Mass Spectrometry to Uncover the Proteome of Cellular Protrusions. Methods Mol. Biol..

[46] Cuella Martin C., Nguyen T.T., Bertin A., Geistlich K., Guyot B., Lefort S., Delay E., Dalverny C., Boissard A., Henry C. (2025). Tunneling Nanotubes Propagate a BMP-Dependent Preneoplastic State 2025. bioRxiv.

[47] Hodneland E., Lundervold A., Gurke S., Tai X.-C., Rustom A., Gerdes H.-H. (2006). Automated Detection of Tunneling Nanotubes in 3D Images. Cytom. Part J. Int. Soc. Anal. Cytol..

[48] Gurke S., Barroso J.F.V., Hodneland E., Bukoreshtliev N.V., Schlicker O., Gerdes H.-H. (2008). Tunneling Nanotube (TNT)-like Structures Facilitate a Constitutive, Actomyosin-Dependent Exchange of Endocytic Organelles between Normal Rat Kidney Cells. Exp. Cell Res..

[49] Moran D.M., Shen H., Maki C.G. (2009). Puromycin-Based Vectors Promote a ROS-Dependent Recruitment of PML to Nuclear Inclusions Enriched with HSP70 and Proteasomes. BMC Cell Biol..

[50] Van Audenhove I., Denert M., Boucherie C., Pieters L., Cornelissen M., Gettemans J. (2016). Fascin Rigidity and L-Plastin Flexibility Cooperate in Cancer Cell Invadopodia and Filopodia. J. Biol. Chem..

[51] Riplinger S.M., Wabnitz G.H., Kirchgessner H., Jahraus B., Lasitschka F., Schulte B., van der Pluijm G., van der Horst G., Hämmerling G.J., Nakchbandi I. (2014). Metastasis of Prostate Cancer and Melanoma Cells in a Preclinical in Vivo Mouse Model Is Enhanced by L-Plastin Expression and Phosphorylation. Mol. Cancer.

[52] Klemke M., Rafael M.T., Wabnitz G.H., Weschenfelder T., Konstandin M.H., Garbi N., Autschbach F., Hartschuh W., Samstag Y. (2007). Phosphorylation of Ectopically Expressed L-Plastin Enhances Invasiveness of Human Melanoma Cells. Int. J. Cancer.

[53] Berg J.S., Derfler B.H., Pennisi C.M., Corey D.P., Cheney R.E. (2000). Myosin-X, a Novel Myosin with Pleckstrin Homology Domains, Associates with Regions of Dynamic Actin. J. Cell Sci..

