# Supplementary material for: Myosin-X Acts Upstream of L-Plastin to Drive Stress-Induced Tunneling Nanotubes
Source: Cells. 2026 Jan 24;15(3):224. doi: 10.3390/cells15030224 (PMC12897106; doi:10.3390/cells15030224)
Supplement: Supplementary file 1 [file cells-15-00224-s001.zip › cells-4086309-supplementary.pdf]

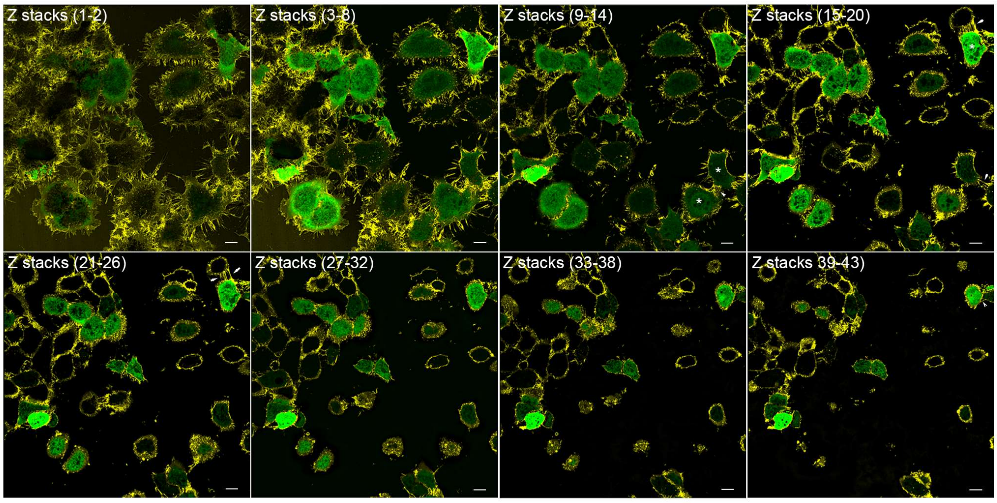

(a)

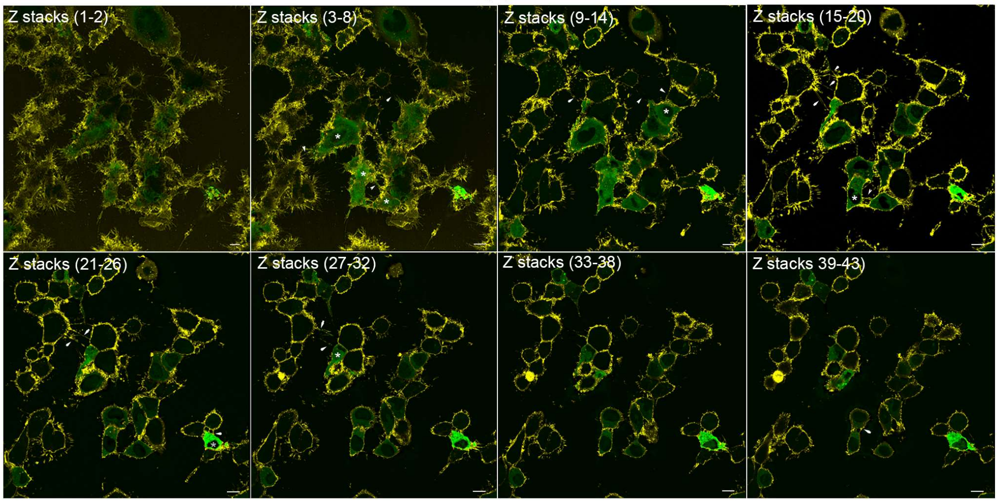

(b)

Figure S1: L-plastin Upregulation Increases the number of cells connected by TNTs. CAD cells transfected with GFP-vector or GFP-L-plastin were imaged and Z-stacks of random fields were acquired. To identify TNTs, Z-stacks are analyzed going from the bottom of the Z-stack (i.e. substratum) to the top of the cells. (**a**) Representative 3D images from the Z-stacks acquired for Figure 2c are shown for the control GFP transfected cells. (**b**) Representative 3D images from the Z-stacks acquired for Figure 2e are shown for the GFP-L-plastin transfected cells.TNTs are highlighted with white arrowheads and transfected cells connected by TNTs are noted with white stars (*). All the experiments were acquired and analyzed under blinded conditions to minimize observer bias. Scale bars = 10 um.

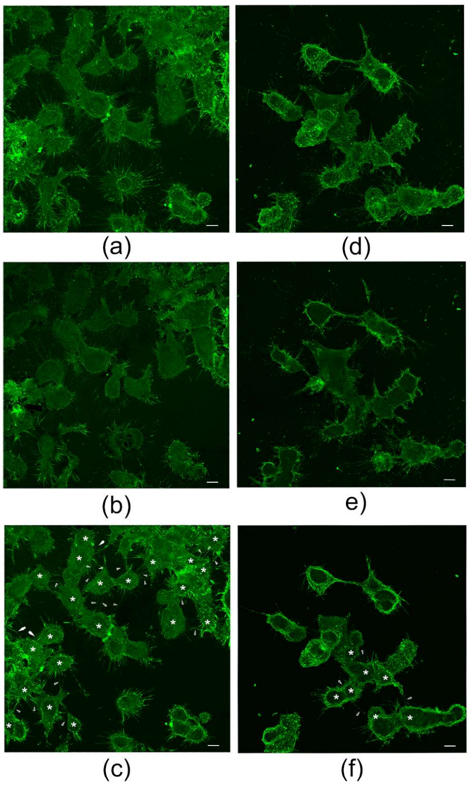

Figure S2: L-plastin downregulation decreases the number of cells connected by TNTs. (**a**) Representative 3D image of control shRNA transduced cells. (**b**) Bottom z-stacks from (a) representing adhering filopodia. (**c**) Top Z-stacks from (a) to identify TNTs (white arrowheads). Cells connected with TNTs are highlighted with a white star (*). (**d**) Representative 3D image of shRNA L-plastin transduced cells. (**e**) Bottom z-stacks from (d) representing adhering filopodia. (**f**) Top Z-stacks from (d) to identify TNTs (white arrowheads). Cells connected with TNTs are highlighted with a white star (*). Scale bars =10 um.

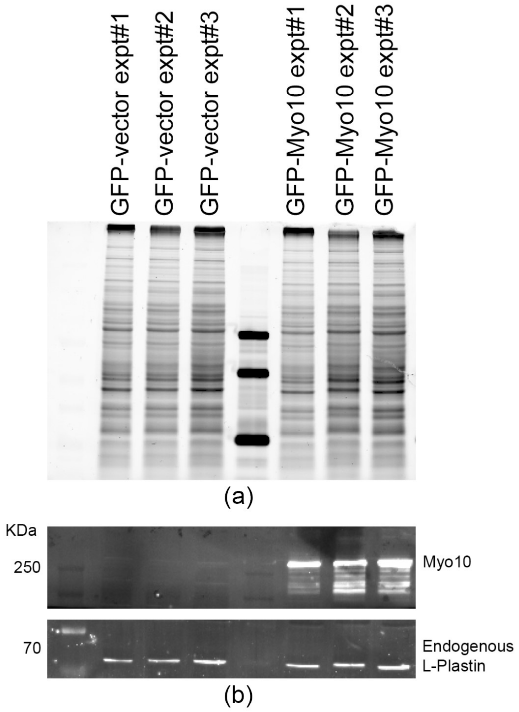

Figure S3: Myo10 expression levels affect L-plastin expression. Three independent experiments were loaded on the gel. The GFP transfected control samples were ran first, followed by the samples transfected with GFP-Myo10 because of the very bright signals from GFP-Myo10 transfection. (**a**) The stain free gel is shown as a loading control. (**b**) Representative Fluorescent Western Blot showing the levels of Myo10 and endogenous levels of L-plastin in these experiments. Lane 1 (GFP-vector expt #1) and lane 4 (GFP-Myo10 expt #1) are the representative lanes juxtaposed in Figure 1 (e) and (f).
